# Changes in Italian Nurses' Genetic Knowledge and Perceptions Over a Decade

**DOI:** 10.1111/nhs.70298

**Published:** 2026-02-01

**Authors:** Lea Godino, Camilla Elena Magi, Mattia Ricco, Daniela Turchetti

**Affiliations:** ^1^ Medical Genetics Unit, IRCCS Azienda Ospedaliero‐Universitaria di Bologna Bologna Italy; ^2^ Department of Health Science University of Florence Florence Italy; ^3^ Dipartimento di Scienze Mediche e Chirurgiche Università di Bologna Bologna Italy

**Keywords:** genetics, knowledge, nurses, questionnaire, survey

## Abstract

Genetic knowledge is increasingly important in nursing, yet often seen as of limited relevance. This study examines Italian nurses' genetic knowledge and perceived relevance, comparing current findings with data from a 2011 survey. A cross‐sectional survey was carried out using a self‐administered questionnaire. A total of 504 nurses (95.1% of individuals logged in) completed the survey. Among those, 78.9% correctly answered at least four of five questions on Genetics knowledge, compared to 60.5% in 2011. There were no significant differences in knowledge by age or academic qualifications. A minority of respondents (26.9%) believed Genetics was highly relevant to the nursing role (26.8% in 2011) and only 8.3% of those reported high confidence in using genetics in their daily work (7.3%). Despite low perceived relevance, 30.6% expressed strong interest in receiving more education to support their practice and 56.1% indicated interest, though not as a high priority, compared to 44.9% and 41.3%, respectively, in 2011. Overall, although knowledge has improved over time, it remains insufficient to support confidence and integration into practice, highlighting the need for targeted genetic education in nursing.

## Key Points

- Studies carried out in 2011‐2012 found Italian nurses had limited genetic knowledge.
- Italian nurses know basic genetics but face challenges in applying it in practice.
- Findings support stronger, practice‐oriented genetics education for Italian nurses.

## 1 Introduction

Genetic and genomic medicine has become an integral component of contemporary healthcare systems, with a rapidly expanding role across prevention, diagnosis, treatment, and risk communication (Murakami et al. 2020). The application of Genetics encompasses the entire healthcare continuum, starting from the preconception period where screening for familial mendelian disorders is crucial (Sagaser et al. 2023); identifying these conditions can facilitate preimplantation genetic diagnosis to prevent their transmission to offspring or can inform prenatal care. Following birth, research is ongoing comparing traditional newborn screening with newborn genome sequencing: preliminary results suggest that genome sequencing identifies actionable conditions missed by traditional methods without causing lasting negative psychosocial impacts (Armstrong et al. 2022; Green et al. 2023).

Genetics is increasingly used also for predicting the risk of common health conditions, including but not limited to cancer and cardiovascular diseases. The field has seen and is going to see significant growth due to decreasing sequencing costs, leading to the current practice of using multi‐gene panel tests of increasing sizes and, in certain cases, whole‐exome or whole‐genome sequencing (Deverka et al. 2023).

As the largest profession in the healthcare system and the first point of contact for patients, nurses are increasingly exposed to genomic data pertinent to patient treatment and need to understand their significance in order to help patients' understanding and proper management. It is therefore crucial for nurses at all academic levels, roles and specialties to be competent in genetics: this need for comprehensive nursing genetic knowledge, skills, and attitudes led to the establishment of minimum competencies in genetics and genomics, ensuring relevance for all nursing professionals (Calzone et al. 2024, 2018a; Chow et al. 2023; Hickey et al. 2018; Skirton et al. 2010; Zureigat et al. 2022).

In 2000, the National Coalition for Health Professional Education in Genetics (NCHPEG) defined core genetic competencies for all healthcare professionals, including nurses. These competencies span 10 key areas, such as basic genetic concepts, family history assessment, the role of genetics in disease prevention and treatment, and awareness of sociocultural and ethical issues.

Since then, the rapid integration of genomics into routine healthcare has substantially expanded the scope and complexity of competencies required of nursing professionals. More recent work has therefore revisited and updated competency frameworks to reflect contemporary practice needs. In particular, Tognetto et al. (2019) proposed updated core competencies in genetics for healthcare professionals through a comprehensive literature review followed by a Delphi process, emphasizing not only knowledge acquisition but also clinical application, communication, ethical decision‐making, and interprofessional collaboration in genetic and genomic care.

Despite the availability of both foundational and updated competency frameworks, international studies highlight persistent gaps in nurses' genetic literacy and their confidence in applying genomic knowledge in practice (Thomas et al. 2023). However, there appears to be a lot of regional variation in how Genetics and Genomics are taught to and applied in clinical practice by nurses (Calzone et al. 2018b; Rehm 2017).

In Italy, two studies conducted between 2010 and 2012 investigated nurses' and midwives' knowledge and perceptions in nursing practice. While 40.4% of nurses correctly answered at least four out of five genetics‐related questions, 62%–63% reported believing that nurses had no role in genetics‐related care (Godino et al. 2013a, 2013b). These studies represent the only Italian evidence specifically addressing nurses' knowledge and perceptions of genetics, as confirmed by a recent scoping review (Dante et al. 2025), and therefore provide a relevant reference point for comparison.

Over a decade from the previous survey, given the increasing relevance of Genetics in healthcare and, consequently, in nurses' practice (Abad and Sur 2022; Aiello 2017; Beery and Williams 2007; Boyd et al. 2017; Buaki‐Sogo and Percival 2022; Chow et al. 2023), we launched a new survey with the aim to explore the current status of Genetics knowledge and perception among Italian nurses.

## 2 Research Aims

This study investigated Italian nurses' perceptions of Genetics in relation to nursing practice, specifically examining their fundamental genetic knowledge, their views on the application of genetics within the profession, and their perceptions of the role of the genetic nurse.

## 3 Methods

### 3.1 Design

A cross‐sectional study was conducted using a self‐administered questionnaire from a previous study (Godino et al. 2013a) and is reported in accordance with the STrengthening the Reporting of OBservational Studies in Epidemiology (STROBE).

### 3.2 Study Setting and Sampling

Participants were recruited through an online questionnaire disseminated via multiple channels, including social media platforms (WhatsApp, Twitter, Facebook, and Instagram), using both institutional channels and the personal social media accounts of the study authors. In addition, registered nurses were recruited through advertisements posted on local websites and social media pages of the nurse registration body, Ordini Professioni Infermieristiche (OPI) and/or sent to respective mailing lists. Nursing Schools were also requested to send advertisements to mailing lists of graduates.

### 3.3 Inclusion and Exclusion Criteria

A sample of Registered Nurses residing in Italy was recruited for the study, with inclusion criteria specifying that each participant was required to be (1) 21–65 years old and (2) registered with the OPI. Exclusion criteria included pre‐registration nursing students and retired practitioners.

### 3.4 Data Collection

Data collection was conducted using a questionnaire uploaded to the Microsoft Form website. The survey was open to respondents between December 1, 2022 and March 31, 2023. The questionnaire reported in Supporting Information titled Survey Questionnaire in our previous paper (Godino et al. 2013a) was used. The questionnaire had 21 items divided into three distinct sections: demographic characteristics (including workplace information); genetic knowledge; and perceptions of the role of the genetic nurse.

The questionnaire includes multiple‐choice items, Likert‐type items assessing perceived relevance and confidence, and open‐ended questions. The genetic knowledge section comprises five multiple‐choice questions, each scored as correct or incorrect, with higher total scores indicating greater genetic knowledge.

### 3.5 Data Analysis

In this cross‐sectional study, data were entered into a dedicated database, arranged by variables and finally analyzed using the Statistical Package for the Social Sciences (SPSS) 28 for Windows. We used descriptive statistical tests to analyze demographic data. The Kruskal–Wallis one‐way analysis of variance by ranks was applied to scale and ordinal variables, the Pearson chi‐squared to nominal variables and Fisher's exact test to dichotomous variables. Two‐tailed *p* values less than 0.05 were considered statistically significant.

### 3.6 Ethical Considerations

The study followed the principles of the Declaration of Helsinki and obtained ethics approval from the Ethics Committee of the University of Bologna (Italy) on November 9, 2022 (approval number 0299217). The participants were informed of the details of the study, including its voluntary nature. Moreover, the participants were informed that they would only be asked to enter data into the questionnaire anonymously, to protect their confidentiality. Any person entering the survey site has the option of leaving without providing any information or after only partially completing it. At the beginning of the survey, respondents were asked to indicate their consent to participate, and they were unable to move on to the questions until they had done so. At the end of the questionnaire, only when the respondent directly pressed the “submit” button did the data get sent to the survey site.

## 4 Results

### 4.1 Characteristics of Participants

The total population eligible to participate in the study comprised all registered nurses in Italy, excluding those who were retired. Of 568 individuals who logged onto the survey site, 550 (96.8%) consented to being involved in the study. The average time spent per each questionnaire was 8 min and 53 s. After excluding respondents who were not Registered Nurses, a total of 540 (95.1%) participants were included in the analysis.

Study participants were mainly females (81.7%) and ranged in age from 21 to 65 years (the majority, 68%, was 45 or younger); 20% (*n* = 119) had registered as nurses within the previous 4 years. The average time of working as a nurse was 15 years (SD = 11.43, range 0–41 years). The characteristics of participants are detailed in Table S1.

As reported in Table S1, 56.0% of respondents reported they did not attend Genetics classes during their degree programs, and 86.7% reported not having received any Genetics education outside of their original nursing program, with significant differences according to age classes: attending Genetics classes during their degree was reported by 63.6% of those aged 21–25 years, 57.5% of those aged 26–30 years, 55.8% of those with age 31–35 years, but only 9.1% of respondents belonging to the age class 56–60 years stated (*p* < 0.001).

### 4.2 Relevance and Confidence

Among participants, 26.9% (*n* = 145) believed Genetics was very relevant to their daily work, slightly more than a third (35.6%, *n* = 192) believed that genetics was quite relevant, 23.1% (*n* = 125) minimally relevant, while 14.4% (*n* = 78) responded that it had no relevance at all.

A summary of the main themes emerging from the open‐ended responses is presented in Table 2, while selected verbatim quotations are reported below to illustrate participants' perspectives. Some respondents who made additional comments were convinced of the relevance, mentioning specific communicative competencies:

The knowledge of pathological conditions (…) allows to have a more complete view of the patient who is affected, so as to improve the quality of care (Respondent 205, male, age range 21–25, score of knowledge 5, clinical area: Geriatrics).

Many of the patients I work with have genetic pathologies and it is often necessary to have a good knowledge of genetics to be able to explain how to deal with the disease (Respondent 414, female, age range 21–25, score of knowledge 5, clinical area: Neonatal Intensive Care).

However, others felt genetics was not applicable within the nursing context:

I don't work in a field requiring knowledge in genetics (Respondent 52, male, age range 21–25, score of knowledge 4, clinical area unknown).

It's physicians' competencies (Respondent 62, female, age range 21–25, score of knowledge 2, clinical area: Neonatal Intensive Care).

I can't see a practical utility of genetics in my daily work (Respondent 197, female, age range 26–30, score of knowledge 5, clinical area: District Nurse).

It's hard to apply genetics in daily care (Respondent 441, male, age range 41–45, score of knowledge 4, clinical area: Gastroenterology).

Little confidence in using Genetics in their daily work was reported by 42.8% (*n* = 231) and only 8.3% (*n* = 45) felt very confident. Their comments reflected this (see Table 2 for a summary of the main themes):

I confidently apply the basic knowledge gained during my studies, but I believe that it is insufficient to provide accurate information about many medical conditions (Respondent 12, female, age range 26–30, score of knowledge 5; clinical area: district nurse).

I would not be able to give much information to a patient in this sense, but I would be able to direct him in those situations in which genetic counseling could be useful (Respondent 22, male, age range 26–30, score of knowledge 4; clinical area: Emergency).

The basic knowledge of genetics acquired during the course of study is not then resumed nor does it have the opportunity to apply it in one's own work context or if one does not have the skills (Respondent 40, male, age range 26–30, score of knowledge 5, clinical area Emergency).

I don't know how to apply genetics knowledge (Respondent 82, female, age range 46–50, score of knowledge 5; clinical area: Oncology).

To date there are no courses in Italy of medical genetics, all the information I have is the result of personal research and questions to the doctors responsible for the service (Respondent 189, female, age range 36–40, score of knowledge 5; clinical area: Oncology).

It fascinates me but I can't be sure about my knowledge of genetics (Respondent 235, female, age range 41–55, score of knowledge 5; clinical area: Nephrology).

Having little knowledge in this field I also have little confidence in using notions (Respondent 405, male, age range 21–25, score of knowledge 3; clinical area: Geriatrics).

There were no significant differences between relevance and confidence when analyzed according to the highest academic qualification of respondents or their knowledge score.

### 4.3 Genetics Education Perceived Needs

Despite most participants not recognizing the relevance of Genetics, 30.6% (*n* = 165) responded they definitely would like more education in Genetics to help them in their daily work, and 56.1% (*n* = 303) also would like more education, but not as a high priority (Table 1).

**TABLE 1:** Perceived relevance, nurse's confidence, and education related to genetics.

Question	Godino et al. 2013b	Current study	Godino et al. 2013b	Current study	Godino et al. 2013b	Current study	Godino et al. 2013b	Current study
	Very *n* (%)		Little *n* (%)		Minimally *n* (%)		No *n* (%)	
How much is genetics relevant to your daily work?	103 (26.8)	145 (26.9)	122 (31.7)	192 (35.6)	122 (31.7)	125 (23.1)	70 (18.2)	78 (14.4)
How confident do you feel about using genetics in your daily work?	28 (7.3)	45 (8.3)	173 (44.9)	231 (42.8)	103 (26.8)	183 (33.9)	43 (11.2)	81 (15.0)
Would you like to have more education in genetics to help you in your daily work?	Definitely		I'd like to, but it isn't a priority		I'm not sure		No	
	173 (44.9)	165 (30.6)	159 (41.3)	303 (56.1)	11 (2.9)	49 (9.1)	3 (0.8)	23 (4.3)

A summary of the main themes emerging from the open‐ended responses regarding perceived educational needs is presented in Table 2, while selected verbatim quotations are reported below to illustrate participants' perspectives. Some of the respondents who wanted further education explained what they wanted to learn in their comments:

How can this knowledge affect my work? How could I put them into practice (Respondent 82, female, age range 46–50, score of knowledge 5; clinical area: Oncology).

Role of the Genetic Nurse (Respondent 157, female, age range 46–50, score of knowledge 4; clinical area unknown).

Not having basic knowledge, I would like to have general information (Respondent 428, female, age range 26–30, score of knowledge 3; clinical area: Intensive Care).

Effective methods of counseling to give a first response to the citizen and then address it accordingly at the right settings (Respondent 541, male, age range 31–35, score of knowledge 5, clinical area: Emergency).

Information regarding genetic diseases, inheritance, being carriers, and the risk of transmitting diseases to one's offspring (Respondent 32, male, age range 36–40, score of knowledge 5, clinical area: Gastroenterology).

(…) about pharmacogenomics (Respondent 23, female, age range 46–50, score of knowledge 3; clinical area: Oncology).

The importance of genetics in oncology (Respondent 23, female, age range 46–50, score of knowledge 3; clinical area: Oncology).

Regarding the relevance of correlation with degenerative diseases (Respondent 29, male, age range 31–35, score of knowledge 4; clinical area: District Nurse).

Correlation between genetics and heart diseases. (Respondent 163, female, age range 46–50, score of knowledge 3; clinical area: Cardiology).

**TABLE 2:** Summary of key themes emerging from open‐ended responses.

Area explored	Main themes identified	Illustrative content (summary)
Perceived relevance of genetics	Genetics as integral to holistic patient care	Genetics perceived as useful for understanding disease mechanisms, improving patient management, and supporting explanations to patients, particularly in areas such as geriatrics and neonatal care
	Limited applicability to nursing practice	Genetics perceived as not relevant in some clinical settings or considered outside the nursing role, sometimes viewed as a physician‐only competency
Confidence in using genetics	Insufficient applied knowledge	Respondents reported having basic theoretical knowledge but lacking the ability to apply genetics in clinical practice or communicate genetic information to patients
	Lack of formal education and training	Limited availability of genetics education during and after nursing education was perceived as a barrier to confidence
	Reliance on informal learning	Genetics knowledge often acquired through self‐study or consultation with physicians rather than structured training
Perceived educational needs	Need for foundational genetic knowledge	Requests for basic information on genetics, inheritance patterns, carrier status, and disease transmission
	Application of genetics to practice	Desire to understand how genetics affects daily nursing work and how to translate knowledge into practice
	Role of the genetic nurse	Interest in clarifying responsibilities and scope of practice of genetic nurses
	Counseling and communication skills	Need for training in patient communication, initial counseling, and referral pathways
	Disease‐specific genetics	Interest in genetics related to oncology, cardiology, degenerative diseases, and pharmacogenomics

A significant difference was observed by perceived relevance: participants with higher perceived relevance of Genetics were more likely to express a strong wish to have more education in Genetics (82/145, 56.6% of those considered genetics as very relevant, vs. 46/192, 24.0% of those considered genetics as little relevant, 24/125, 19.2% of those considering genetics as minimally relevant and 13/78, 16.7% of those considered genetics as no relevant; *p* < 0.001). Similar results were found by confidence (24/45, 53.3% of those feeling very confident, vs. 84/231, 36.4% of those feeling little confident, 44/183, 24.0% of those feeling minimally confident and 13/81, 16.0% of those feeling not confident at all; *p* < 0.001). Moreover, attending genetics' education outside the original nursing program was significantly correlated with confidence (17/43, 39.5% of those feeling very confident, versus 34/221, 15.4% of those feeling little relevant, 14/176, 8.0% of those considered genetics as minimal relevant and 4/77, 5.2% of those considered genetics as no relevant; *p* < 0.001).

### 4.4 Knowledge of Genetics

Out of 540 participants, 38.3% (*N* = 138) correctly responded to all five questions and 40.6% (*n* = 146) four questions on knowledge of genetics (mean = 4.10 SD = 0.91 range = 0–5) (Table 3). The detailed knowledge questions and scores are shown in Table S2. A statistically significant correlation (*p* = 0.044) was found between genetics' knowledge score and attending a genetic course during the degree programs: nurses are more likely to obtain score 4–5 than 0–3 (137/164, 52.1% vs. 27/164, 38.0%). Instead, there were no significant differences between knowledge scores when analyzed according to age of respondents, highest academic qualification of respondents, relevance, or confidence.

**TABLE 3:** Registered nurses' genetic knowledge score.

Score	Godino et al. 2013a	Current study
0	3 (0.9)	1 (0.3)
1	16 (4.9)	3 (0.8)
2	37 (11.4)	16 (4.4)
3	72 (22.2)	56 (15.6)
4	121 (40.4)	146 (40.6)
5	65 (20.1)	138 (38.3)
Total	324	360

### 4.5 Roles of the Genetic Nurse Specialist

As shown in Table 4, according to the majority of participants, trained genetic nurses could undertake four out of nine roles listed. Obtaining informed consent was considered their own role in 49.3% of respondents. The majority of participants expressed as “possible” drawing a family tree. Taking a family history, explaining the genetics test, and the result of it were considered also as possible roles (49.3%, 46.9%, and 46.5% respectively).

**TABLE 4:** Respondents' opinions about the roles of genetic nurses.

	Answers *n* (%)					
	Definitely		Possibly		Never	
	Godino et al. 2013a	Current study	Godino et al. 2013a	Current study	Godino et al. 2013a	Current study
Genetic Nurse						
Take a family history	273 (86.9)	256 (47.4)	36 (11.5)	266 (49.3)	5 (1.6)	18 (3.3)
Draw family tree	156 (52.9)	201 (37.2)	129 (43.8)	290 (53.7)	10 (3.3)	49 (9.1)
Find out about the patient's concerns	271 (87.1)	390 (72.2)	40 (12.9)	136 (25.2)	0 (0.0)	14 (2.6)
Explain the genetics test	189 (62.1)	146 (27.0)	94 (30.1)	253 (46.9)	21 (6.8)	141 (26.1)
Obtain the informed consent	164 (55.0)	266 (49.3)	95 (31.8)	161 (29.8)	39 (13.2)	113 (21.0)
Take the blood sample for a genetic test	287 (93.2)	431 (79.8)	21 (6.8)	99 (18.3)	0 (0.0)	10 (1.9)
Explain the result of a genetic test	89 (30.6)	224 (41.5)	113 (40.0)	251 (46.5)	87 (29.4)	65 (12.0)
Offer the patient psychological support	191 (62.0)	360 (66.7)	111 (36.0)	171 (31.7)	6 (2.0)	9 (1.6)
Provide follow up to the family	198 (64.7)	369 (68.3)	98 (32.0)	163 (30.2)	10 (3.3)	8 (1.4)

## 5 Discussion

This study aimed to assess Italian nurses' genetic knowledge and perceptions of Genetics in relation to nursing practice. Since nurses responded from every part of Italy, this study may allow to draw some conclusions regarding Registered Nurses in this nation. In the previous study conducted by the same research group, it came to light that nurses had an adequate understanding of Genetics (Godino et al. 2013a). In fact, 20.1% of nurses properly responded to all five questions, and another 40.4% correctly answered four questions. In the present study, a further improvement was found, as the proportion of nurses who properly responded to all five questions increased by 18.2% points. This is in contrast with findings from a recent scoping review revealing that nurses have inaccuracies in their understanding of Genetics and Genomics (Thomas et al. 2023). This includes a lack of comprehension of essential concepts like the difference between germline and somatic (Hines‐Dowell et al. 2024) or the use of evidence‐based pharmacogenomics (Fulton et al. 2024), which poses obstacles to the integration of Genetics into clinical practice (Thomas et al. 2023). Similar findings emerged from a 2025 study conducted in New Zealand, which showed that lack of knowledge and persistent misconceptions were also observed among preregistration nurses, highlighting the urgent need to ensure understanding of basic concepts and to include more nursing‐specific genetic content in curricula (Cao et al. 2025; Lim et al. 2025; Parviainen et al. 2023). A reason for the discrepancies between our results and those studies may be that in our study ascertainment of Genetics knowledge only relied on a few basic questions, while in the other studies a more in‐depth assessment was performed. Whereas there is increasing consensus on the fact that nurses should receive education on basic principles of genomics, precision medicine, and precision health, as well as nurses' roles, real‐world nursing applications, and the ethical, legal, and social consequences of genetic concepts, 56% of the participants in this study reported not having attended any Genetics class during the Nursing School: although the proportion was lower among younger participants, it was yet suboptimal (36.4% in those aged 21–25).

Recent developments in Genetics have the potential to aid in the identification of individuals at risk in many settings such as Huntington Disease care (Baker et al. 2013) or cardiovascular disorders (Mital et al. 2016). Moreover, several learning needs, including the interpretation of genomic data, clinical decision‐making based on genetic findings, and effective patient communication and counseling, were identified for oncology nurses to integrate genomic testing into their clinical practice (Rahmani et al. 2014). Nevertheless, in our survey, open statements highlighted that the perceived transferability of Genetics knowledge in daily work was scarce even for nurses working in those areas where Genetics is crucial to clinical practice, including Oncology, Pediatrics, and Cardiology, suggesting that increased exposure to Genetics in these departments does not necessarily correlate with greater awareness.

Consistently, only 8.3% of participants reported high confidence in using Genetics in their daily work. The findings on nurses' perceptions of the relevance of their role in genetic healthcare confirm those of our previous study (Godino et al. 2013a) and those of other studies in other countries (Thomas et al. 2023). Nurses lack skills in the application of Genetics and Genomics to patient care (Camak 2016). Similar results were found in a recent qualitative Australian study that highlighted that nurses and midwives were aware of the need to upgrade their Genomics skills but were still unsure of how it related to their daily practice (Laaksonen et al. 2022).

Beyond the use of Genetics by nurses in different settings, the study explored the awareness of the role of trained Genetic Nurses. If compared to our previous study, tasks commonly delivered by trained genetic nurses were correctly recognized by fewer participants, suggesting that current participants had a more restrictive view of responsibilities that a nurse should take. This may be influenced by the increasing attitudes toward defensive medicine, where healthcare providers adopt a cautious approach to minimize legal risks (Strobel et al. 2023) and adhere strictly to well‐established and legally unambiguous roles. Another possible explanation is the fear for nurses to take additional workload in an era where work dissatisfaction is increasingly common, and many professionals feel overwhelmed and decide to quit working (Al Zamel et al. 2020; Lyu et al. 2024). However, it is important to note that in Italy this professional figure is still not well‐defined, which may further limit the perception of the actual role of a genetic nurse. In fact, despite the significant demand for genetic services, in Italy, there is a heterogeneous panorama concerning the role and activities of the nurse who works in genetics services (Godino, Magi, et al. 2025; Godino, Ambrosini, et al. 2025).

### 5.1 Limitations of the Work

This study has several limitations that should be acknowledged. First, the use of an online survey may have introduced selection bias, as only social media users could learn about it and nurses with an interest in Genetics were more likely to participate, thus findings may not reflect the attitudes of the whole nurse community in Italy. Secondly, the use of a four‐point Likert scale consisting of “very,” “little,” “minimal,” and “no” may have introduced a potential bias: the second point on the scale, “little,” was not positively framed, possibly leading to an overestimation of negative responses. This design choice could have skewed the results toward a more negative interpretation. Finally, comparisons between data from previous and current studies were shown, but no statistical analyses were performed. However, since comparison was not a primary aim of the study, we feel this does not weaken the results of the study while providing some insights on evolution over time.

## 6 Conclusions

It is essential for all nurses to have a sufficient level of genetic competency. The findings of this study suggest that although there is a growing trend in genetics knowledge among nurses, it is not sufficient to enable them to confidently apply it in their everyday work. The main reason is the lack of adequate training focused on developing the necessary skills to enable nurses to apply genetics knowledge more confidently. Nurses, who are pivotal in assisting patients in managing health and illness, must possess genetic knowledge to educate and deliver comprehensive care to individuals as they make important genetic decisions. In conclusion, we underline the need for enhanced education and training for nurses to improve care for individuals with genetic disorders.

### 6.1 Relevance for Clinical Practice

Nurse educators should advocate for a more comprehensive integration of Genetics into standard nursing education and practice. They should emphasize that Genetics education needs to be closely linked to the experiences of families and patients to help nurses effectively apply theoretical knowledge to their clinical practice. Despite international efforts, the profession of genetic counselors and nurses is not formally recognized in numerous countries, including Italy (Abacan et al. 2019; Paneque et al. 2016), which makes it crucial to promote genetic literacy across the entire nursing profession. As Genetics increasingly shapes clinical care, nurses need to feel prepared and confident in supporting individuals and families along these complex pathways. In this regard, innovative approaches, such as mobile‐based learning, have proven effective in enhancing nurses' genetic knowledge, as demonstrated by a recent quasi‐experimental study in Turkey using WhatsApp‐based training (Ceylan et al. 2025). Such tools can help overcome traditional barriers to continuing education and should be considered in institutional strategies. Our findings will allow institutions to develop strategies at multiple levels to improve Genetic knowledge.

## Author Contributions

**Lea Godino:** conceptualization, data curation, formal analysis, investigation, methodology, project administration, visualization, and writing – review and editing. **Camilla Elena Magi:** data curation, investigation, and writing – original draft. **Mattia Ricco:** formal analysis and writing – review and editing. **Daniela Turchetti:** conceptualization, data curation, methodology, supervision, writing – review and editing.

## Funding

The authors have nothing to report.

## Ethics Statement

The study was approved by the Ethics Committee of the University of Bologna on November 9, 2022 (approval number 0299217).

## Consent

The authors have nothing to report.

## Conflicts of Interest

The authors declare no conflicts of interest.

## Supporting information


**Table S1:** nhs70298‐sup‐0001‐Tables.docx.
**Table S2:** nhs70298‐sup‐0001‐Tables.docx.

**Data S1:** nhs70298‐sup‐0002‐Strobe checklist.docx.

## Data Availability

The data that support the findings of this study are available from the corresponding author upon reasonable request.
