# Supplementary material for: Changes in Italian Nurses' Genetic Knowledge and Perceptions Over a Decade
Source: Nurs Health Sci. 2026 Feb 1;28(1):e70298. doi: 10.1111/nhs.70298 (PMC12861578; doi:10.1111/nhs.70298)
Supplement: Supplementary file 1 — Table S1: nhs70298‐sup‐0001‐Tables.docx. Table S2: nhs70298‐sup‐0001‐Tables.docx. [file NHS-28-e70298-s002.docx]

**SUPPLEMENTARY FILE**

- **Table S1 Characteristics of participants**

|  | n (%) |
| --- | --- |
| Gender  **Female**  Male  Prefer not to say | **441 (81.7)**  96 (17.8)  3 (0.6) |
| Age  21-25 year  **26-30 year**  31-35 year  36-40 year  41-45 year  46-50 year  51-55 year  56-60 year  61-65 year | 80 (14.8)  **97 (18.0)**  82 (15.2)  54 (10.0)  54 (10.0)  72 (13.3)  56 (10.4)  35 (6.5)  10 (1.9) |
| Highest qualification in nursing  Diploma  **Degree**  Master I degree  Specialist degree  Master II degree  Doctorate | 70 (13.0)  **234 (43.3)**  154 (28.5)  70 (13.0)  7 (1.3)  5 (0.9) |
| Region of Nursing qualification  Abruzzo  Basilicata  Calabria  Campania  Emilia Romagna  Friuli Venezia Giulia  Lazio  Liguria  Lombardia  **Marche**  Molise  Piemonte  Puglia  Sardegna  Sicilia  Toscana  Trentino Alto Adige  Umbria  Veneto | 13 (2.4)  4 (0.7)  3 (0.6)  15 (2.8)  128 (23.7)  2 (0.4)  22 (4.1)  13 (2.4)  50 (9.3)  **158 (29.3)**  2 (0.4)  13 (2.4)  20 (3.7)  5 (0.9)  21 (3.9)  46 (8.5)  3 (0.6)  7 (1.3)  15 (2.8) |
| Primary focus of practice  **Patient care**  Education  Administration/Management  Research  Other  Skipped question | **440 (81.5)**  14 (2.6)  44 (8.1)  14 (2.6)  28 (5.2)  0 (0.0) |
| Area in which you work  Angiology / Haematology  Cardiology / Coronary care  Emergency  Endocrinology  Gastroenterology  Gynaecology/Obstetrics  Hospital hygiene  Infectious diseases  Intensive Care  Management  Medicine/Geriatrics  Mental health  Neonatology/ Neonatal Intensive Care  Neurology  Neuropsychiatric medicine  Oncology  Operating room  Ophthalmology  Orthopaedic  Paediatrics  Physical and Rehabilitation Medicine  **Primary care / District nurse**  Psychiatric medicine  Rare disease center  Renal medicine  Respiratory medicine  Surgery  University Education  Urology / Andrology  Other  Not applicable | 11 (2.0)  19 (3.5)  37 (6.9)  3 (0.6)  14 (2.6)  3 (0.6)  8 (1.5)  10 (1.9)  40 (7.4)  8 (1.5)  65 (12.0)  11 (2.0)  5 (0.9)  7 (1.3)  3 (0.6)  12 (2.2)  30 (5.6)  2 (0.4)  13 (2.4)  7 (1.3)  10 (1.9)  **72 (13.3)**  5 (0.9)  7 (1.3)  18 (3.3)  18 (3.3)  12 (2.2)  6 (1.1)  4 (0.7)  12 (2.2)  41 (7.6) |
| Region of work  Abruzzo  Basilicata  Calabria  Campania  **Emilia Romagna**  Friuli Venezia Giulia  Lazio  Liguria  Lombardia  Marche  Molise  Piemonte  Puglia  Sardegna  Sicilia  Toscana  Trentino Alto Adige  Umbria  Veneto | 3 (0.6)  1 (0.2)  3 (0.6)  11 (2.0)  **168 (31.1)**  3 (0.6)  14 (2.6)  13 (2.4)  51 (9.4)  156 (28.9)  2 (0.4)  14 (2.6)  11 (2.0)  4 (0.7)  18 (3.3)  41 (7.6)  9 (1.7)  5 (0.9)  13 (2.4) |

| Item |  | Answers n (%) | | |
| --- | --- | --- | --- | --- |
|  | n | Correct | Incorrect | Don’t know |
| What is the relationship between a gene and a chromosome?  A gene includes many different chromosomes  One gene equals one chromosome  Chromosome are converted to gene during meiosis  A chromosome includes many different genes (*) | 540 | 370 (68.5) | 96 (17.8) | 74 (13.7) |
| What is the normal human male karyotype?  46,XX  46,XY (*)  44,XX  44,XY | 540 | 365 (67.6) | 139 (25.7) | 36 (6.7) |
| What is the definition of a mutation?  The location of a gene on a chromosome  A change in a gene that can be harmful, beneficial, or have no effect (*)  A group of 3 RNA bases  A variation in the DNA shared by a large percentage of the population | 540 | 443 (82.0) | 65 (12.0) | 32 (5.9) |
| What is the cause of Down syndrome?  Trisomy of chromosome 21 (*)  Trisomy of chromosome 18  Monosomy of chromosome 21  Monosomy of chromosome 18 | 540 | 518 (95.9) | 18 (3.3) | 4 (0.7) |
| Which of the following statements is true.  Men cannot inherit a gene fault that causes breast cancer.  Men can inherit a gene fault that causes breast cancer. (*)  Men can inherit a gene fault that causes breast cancer, but cannot pass it to their children. | 540 | 302 (55.9) | 117 (21.7) | 121 (22.4) |

- **Table S2** Registered nurses’ genetic knowledge
